# Simulated outcomes for durotomy repair in minimally invasive spine surgery

**DOI:** 10.1038/s41597-023-02744-5

**Published:** 2024-01-10

**Authors:** Alan Balu, Guillaume Kugener, Dhiraj J. Pangal, Heewon Lee, Sasha Lasky, Jane Han, Ian Buchanan, John Liu, Gabriel Zada, Daniel A. Donoho

**Affiliations:** 1 Department of Neurosurgery, Georgetown University School of Medicine, 3900 Reservoir Rd NW, Washington, D.C. 20007 USA; 2 Department of Neurological Surgery, Keck School of Medicine of University of Southern California, 1200 North State St., Suite 3300, Los Angeles, CA 90033 USA; 3 University of Southern California, 3709 Trousdale Pkwy., Los Angeles, CA 90089 USA; 4 Department of Neurosurgery, Children’s National Hospital, 111 Michigan Avenue NW, Washington, DC 20010 USA

**Keywords:** Preclinical research, Medical research, Health care

## Abstract

Minimally invasive spine surgery (MISS) is increasingly performed using endoscopic and microscopic visualization, and the captured video can be used for surgical education and development of predictive artificial intelligence (AI) models. Video datasets depicting adverse event management are also valuable, as predictive models not exposed to adverse events may exhibit poor performance when these occur. Given that no dedicated spine surgery video datasets for AI model development are publicly available, we introduce Simulated Outcomes for Durotomy Repair in Minimally Invasive Spine Surgery (SOSpine). A validated MISS cadaveric dural repair simulator was used to educate neurosurgery residents, and surgical microscope video recordings were paired with outcome data. Objects including durotomy, needle, grasper, needle driver, and nerve hook were then annotated. Altogether, SOSpine contains 15,698 frames with 53,238 annotations and associated durotomy repair outcomes. For validation, an AI model was fine-tuned on SOSpine video and detected surgical instruments with a mean average precision of 0.77. In summary, SOSpine depicts spine surgeons managing a common complication, providing opportunities to develop surgical AI models.

## Background & Summary

Surgical videos offer valuable information on surgical pathology, surgical decision-making, instrumentation, and surgeon technical skills. Evaluating surgical videos can enhance surgical workflows by providing decision-assistance functions to surgeons, can facilitate automated video reviews to improve surgical training, or allow quantitative analysis of intraoperative events to enhance surgical research^1–3^. Across these surgical specialties, computer vision (CV) and deep learning methods have enabled outcome prediction using intraoperative tool movements and achieved human expert ability to predict actions and surgical skill from operative video^4–23^. To this end, various groups in general surgery, urology, and neurosurgery have leveraged artificial intelligence to facilitate the acquisition, processing, and analysis of surgical videos^1–3,20,24,25^.

However, before applying AI models to the clinical setting, datasets of surgical videos must be constructed to support their development. The curation and open-access publication of video datasets is a critical prerequisite step to the development of artificial intelligence techniques for surgical operative video^4,5,20,22,26,27^. Unfortunately, the current landscape of surgical video analysis is fragmented across different specialties and research groups, with limited public access to many of the databases^5,27–32^. While other specialties have amassed large datasets of surgical videos, neurosurgery and spine surgery, in particular, lag behind^4,5,21,23,28,33^.

At present, there is no video dataset optimized for machine learning applications in spine surgery, creating a critical roadblock for progress in the field^20,27,34,35^. A growing fraction of the 1.6 M spine surgeries performed every year generate video data, creating a potential corpus of video data that may improve patient outcomes and surgeon performance^35,36^.

An ideal surgical video dataset in spine surgery would encapsulate the spectrum of surgical care, including heterogenous surgical video, complications, and non-routine actions^12,20,27,37^. The development of deep learning technologies as applied to surgical video could eventually assist surgeons in managing rare complications, facilitate trainee skill acquisition in the era of duty-hour restrictions, or predict patient outcomes more accurately than current models, which rely largely on past medical history or radiographic data^20^. While the end-use and clinical application of AI models in neurosurgery remains debated, the initial development of high-quality models requires prodigious data - in this case, surgical video.

To address this gap in the literature, we publish the Simulated Outcomes Following Minimally Invasive Simulated Spine Surgery (SOSpine) surgical video dataset to address these needs. SOSpine depicts neurosurgeons managing a MISS durotomy repair with simulated cerebrospinal fluid leak in a perfused cadaveric setting. SOSpine is the first dedicated video dataset with corresponding outcomes and instrument annotations in spine surgery.

## Methods

### Data collection

We recruited neurosurgical trainees above 18 years of age across a spectrum of experience and familiarity with MISS. All trainee participants provided informed consent to participate, and the original study was exempted from full review by the University of Southern California Institutional Review Board (Proposal #HS-21-00694). The participants were given a pre-test to determine prior MISS experience and confidence levels in performing a CSF leak repair. The participants were allowed to practice their technique once before the study trials, and they later performed the durotomy repair task in three successive trials^37^. Detailed methodology, validation, and results of the simulation have previously been published^37,38^. Briefly, a laminectomy was performed in a fresh cadaver and a 12-gauge arterial catheter was inserted into the subdural-subarachnoid space to reconstitute CSF pressures^37,38^. Subsequently, the thoracolumbar spine was exposed and a 22 mm METRx (Medtronic Sofamor Danek, Medtronic Inc, Dublin, Ireland) tubular retractor was positioned at a new spinal segment for each surgeon. A 1 cm durotomy was created and CSF flow was started. Participants were asked to perform the durotomy closure under surgical loupe magnification using fine tip tissue forceps, Castroviejo needle holders and 6-0 Prolene sutures. The time to complete a primary suture repair, using an interrupted suture technique, was recorded, and the dura was pressurized to simulate a Valsalva maneuver. Success was defined by a neurosurgeon as the absence of visible CSF leak following Valsalva maneuver. Visible CSF leak was defined as extravasation of clear fluid from the durotomy repair site based on judgement of the trained expert. In addition to intraoperative video of the participant trials, de-identified task outcomes (time, task success) and demographic data (post-graduate year, prior experience with MISS, number of prior cases) were recorded^37^. All data records presented in this dataset are derived through the aforementioned study procedures. This study was approved by the Institutional Review Board at the University of Southern California and followed appropriate ethical and professional guidelines.

### Data clean-up, annotation, and quality control

**Fig. 1:**
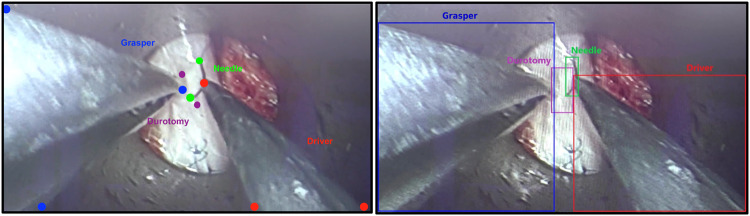
Sample tooltip and bounding box instrument annotations.

Original videos were recorded at a resolution of 1920 × 1080 pixels at 30 frames per second (fps), then down-sampled to 1 fps using FFmpeg while maintaining the same resolution^9^. Trained annotators, comprising of medical and undergraduate students, labeled all tools present in each frame of video using tooltip annotations consisting of needle tip, needle base, needle driver tip(s) and base, grasper tip(s) and base, nerve hook tip and base, and durotomy ends. Tooltip annotations were defined by a single-point bounding box such that the top-left and bottom-right coordinates are identical. Experienced study team members, consisting of senior medical students (DJP, GK), performed quality control and modified tooltip annotations as appropriate. Rectangular bounding boxes were programmatically generated by finding the minimum and maximum of the *x* and *y* coordinates of the tooltip annotations for each instance of each tool. Tool instances with only one tooltip annotation, occurring in the setting of partial obscuration of the tool by other objects in the field of view, were given a square-shaped bounding box by expanding the singular annotation by 50 pixels in all four directions. Annotation coordinates were then compiled into comma-separated value (CSV) files for analysis. A sample image from the SOSpine dataset is shown in Fig. 1 with the associated tooltip annotations.

### Dataset validation model development

For CV analysis, SOSpine trials were randomly divided into the training and testing subsets such that the training set contained approximately 80% of all frames and the testing set contained the remaining 20%. Images from a single trial were all assigned to the same subset to avoid data leakage. The associated annotations used for CV analysis consisted of surgical instrument bounding box coordinates. The training subset contained 12,866 images while the testing subset contained 2,828 images.

YOLOv4 (you only look once version 4) is a one-stage object detection algorithm that implements a 53-layer deep neural network architecture (CSPDarknet53) and additional layers to apply data augmentation and extract potential objects from an image^39–41^. A YOLOv4 model, pre-trained on the Microsoft COCO (Common Objects in Context) image dataset, was further trained on the SOSpine training set images with the following parameters: batch size of 64, a network width and height of 608, and learning rate of 0.001^39–41^. The Amazon Web Services EC2 service was used to train the algorithm in the cloud with a NVIDIA T4 graphics processing unit for a total of 16,000 iterations/batches (79.6 epochs).

### Statistical analysis/model performance validation

For CV model performance analysis, the trained YOLOv4 model was used to detect surgical instruments in the testing subset data. All instrument bounding boxes with a detection confidence greater than 0.05 were collected and compiled into a CSV file. The instrument detections were used to create precision-recall curves for each surgical tool and calculate the average precision (AP) for each tool using the area-under-the-curve (AUC) method. Detected instruments were compared to ground truth annotations using an IoU threshold of 0.50^4,9^.

**Fig. 2:**
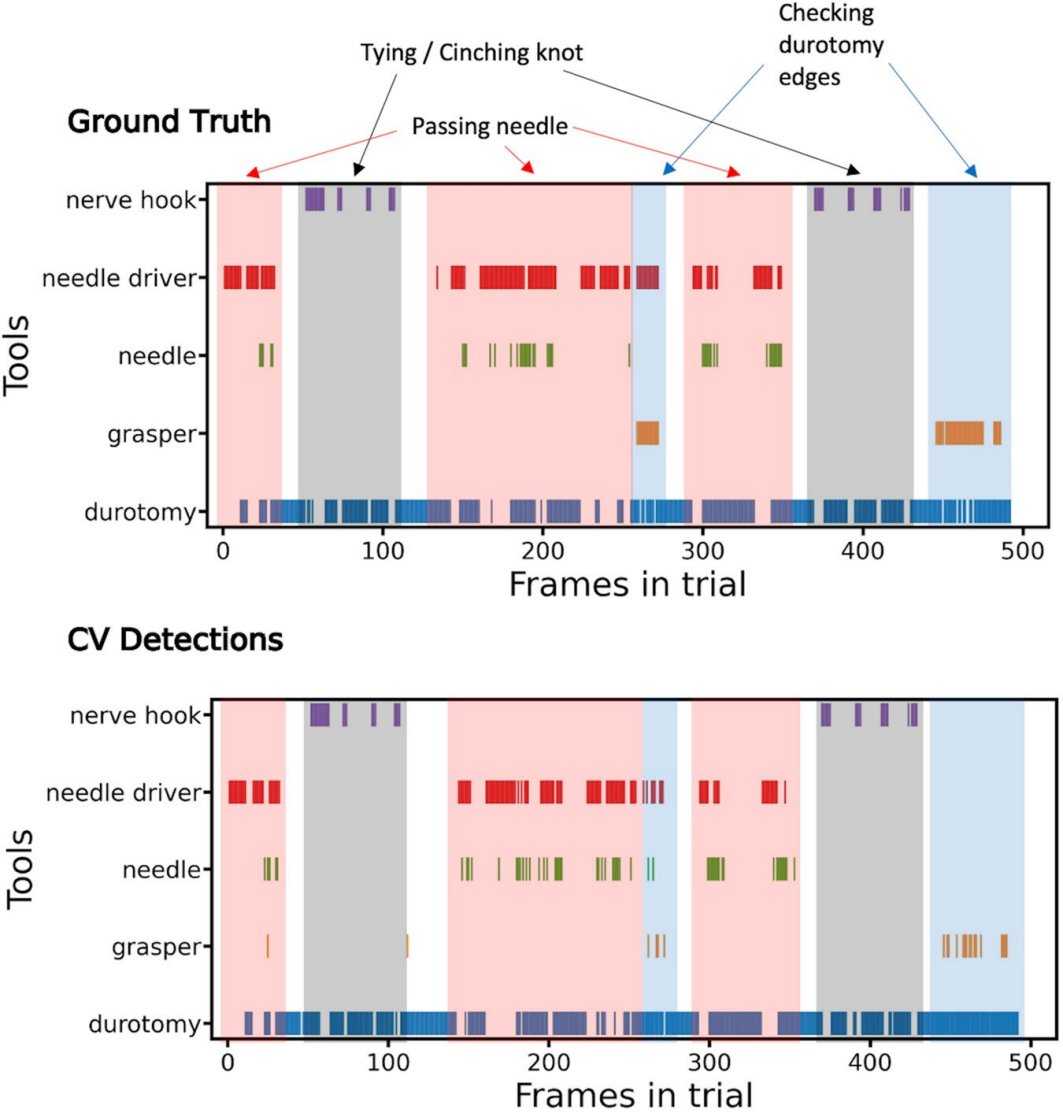
Tool presence comparison between ground-truth and CV detections for a selected SOSpine trial. Surgical actions are highlighted in red, blue, and grey.

In addition, surgical actions within procedures were manually identified by examining patterns in instrument annotations and trial video footage (Fig. 2). Two portions of time that exclusively contained the nerve hook and durotomy, with no other instruments, were referred to as “suture tying.” Similarly, passing the needle through the durotomy (i.e., “taking a bite”) was also identified by areas that contain needle driver, needle, and durotomy together.

## Data Records

We followed the NeurIPS 2021 Code and Data Submission Guidelines for dataset publishing to ensure accessibility^42^. Following these guidelines, the public SOSpine dataset has a Digital Object Identifier (DOI), version tracking, was placed in a repository that ensures long-term preservation, is publicly available for download, and is licensed under a Creative Commons Attribution 4.0 International License through the *FigShare* website^43^. The SOSpine dataset can be found at the following URL: https://figshare.com/projects/Simulated_Outcomes_for_Durotomy_Repair_in_Minimally_Invasive_Spine_Surgery_SOSpine_/142508.

The SOSpine dataset contains 24 recorded and annotated video trials from 8 unique neurosurgeons of varying experience with MISS. 37.5% of surgeons successfully closed the durotomy on their initial attempt, with an average time of 727 seconds to completion. This increased to 87.5% on the third attempt, with a decreasing average time of 424 sec. 17/24 trials were successful with a mean time to closure of 600 seconds. Overall, 62.5% of surgeons failed the initial trial, 12.5% failed trial two, and 12.5% failed trial three. 87.5% of surgeons succeeded in their second and third attempts at durotomy closure. One surgeon with no prior MISS experience failed all three trials.

Video from 24 trials (15,698 frames) underwent 53,238 instrument tip annotations. An additional 21,371 bounding box annotations were created from tooltip annotations (Table 1). The overall cost for storage, annotation, and quality control of the dataset was approximately $2,000.

**Table 1:** Breakdown of SOSpine Video Dataset instrument instances, training, and testing sets.

	All Videos	Training	Testing
Instrument			
Grasper	2,375	1,683	692
Needle Driver	4,238	3,400	838
Nerve Hook	1,200	1,023	177
Non-instrument			
Durotomy	12,141	9,917	2,224
Needle	1,417	1,165	252

The dataset presented here can be found on FigShare and descriptions of the individual files can be found below.

Dataset summary:

Number of surgeons: 8

Number of trials with outcomes data: 24

Number video annotated trials: 24

Number of annotated frames: 15,698

Total tooltip annotations: 53,238

Total bounding box annotation: 21,371

Number of tooltips annotated:

Needle Tip: 1,571

Needle Base: 347

Needle Driver Tip: 4,544

Needle Driver Base: 9,371

Grasper: 8,478

Nerve Hook: 4,648

Durotomy: 24,279

Number of generated bounding boxes:

Needle: 1,417

Needle Driver: 4,238

Grasper: 2,375

Nerve Hook: 1,200

Durotomy: 12,141

### frames.zip

Zip file that contains all video frames, as .jpeg files.

Record: frames.zip.June2022. 10.6084/m9.figshare.20201636.v1^44^.

### sospine_tool_tips.csv

Each row in this file corresponds to a single tooltip annotation in a single frame. If a frame has multiple annotated tools, then there will be a row for each tool in view. If a frame has no tools in view, then there will be a single row for this frame where th e first column contains the frame file name, and the remaining columns are empty. For the coordinate values, the top left corner of the image is considered the origin (0, 0). The coordinates are integer pixel values.

Record: sospine_tool_tips.csv.June2022. 10.6084/m9.figshare.20171135.v1^45^.

trial_frame (column 1) - file name beginning with the trial name and ending with frame number

x1 (column 2) - The left x coordinate

y1 (column 3) - The top y coordinate

x2 (column 4) - right x coordinate

y2 (column 5) - The bottom y coordinate

label (column 6) - The tool tip label. Can be one of 7 labels: needle tip, needle base, durotomy, nerve hook, grasper, needle driver tip, or needle driver base

### sospine_bbox.csv

This file contains bounding boxes generated programmatically using the tooltip annotations previously described in this dataset release. Each row in this file corresponds to a single tool bounding box annotation in a single frame. If a frame has multiple annotated tools, then there will be a row for each tool in view. If a frame has no tools in view, then there will be a single row for this frame where the first column contains the frame filename, and the remaining columns are empty. Bounding boxes were generated by finding the minimum and maximum of the x and y coordinates for all points annotated for a given tool to create a rectangular bounding box. Tools with only one tooltip annotation were simply given a square-shaped bounding box by expanding the singular annotation by 50 pixels in either direction. For the coordinate values, the top left corner of the image is considered the origin (0, 0). The coordinates are integer pixel values.

Record: sospine_bbox.csv.June2022. 10.6084/m9.figshare.20171129.v1^46^.

trial_frame (column 1) - Frame file name beginning with the trial name and ending with frame number

x1 (column 2) - The left x coordinate

y1 (column 3) - The top y coordinate

x2 (column 4) - The right x coordinate

y2 (column 5) - bottom y coordinate

Label (column 6) - The bounding box label. Can be one of 5 labels: needle, durotomy, nerve hook, grasper, or needle driver[e]

### sospine_outcomes.csv

This file contains the participant level demographics data for participating surgeons, results of the repair trials, and information about the associated videos. Missing values indicate either unavailable participant data or unavailable video footage of those trials.

Record: sospine_outcomes.csv.June2022. 10.6084/m9.figshare.20171132.v1^47^.

Trial ID (column 1) - Trial ID that refers to the surgeon and attempt number. S# denoting surgeon and A# denoting the attempt.

Length (column 2) - Length of the video in minutes and seconds.

Postgraduate year (column 3) - Participant’s year along neurosurgery residency

Prior experience with MISS (column 4) - Whether participant had experience with minimally invasive spine surgery cases in the past

Number prior cases (column 5) - Number of minimally invasive spine surgery (MISS) cases that the participant has been involved with prior to the SOSpine trials

Time for repair (column 6) - Number of seconds needed to complete the durotomy repair procedure. Defined from the time from initial instrument contact with dura to the time at which instruments exited the field on securing the final knot

Leak At 40 mmHg (column 7) - Presence of a Cerebrospinal Fluid (CSF) leak after completion of the durotomy repair and pressurizing the system to 40 mm Hg.

Total Frames at 1 FPS (column 8) - Number of video frames from the trial that are included in this dataset within the frames.zip file.

### SOSpine.zip

This zip file contains the figure generation and technical validation Python script in addition to SOSpine dataset files as described above excluding frames.zip.

Record: 10.6084/m9.figshare.22341523.v1^43^.

## Technical Validation

Surgical video datasets such as SOSpine offer a set of challenging visual data that can be used to train and subsequently test computer vision algorithms. We also provide benchmark performance and dataset validation using the standard AI task of object detection, in this case, surgical instruments.

**Fig. 3:**
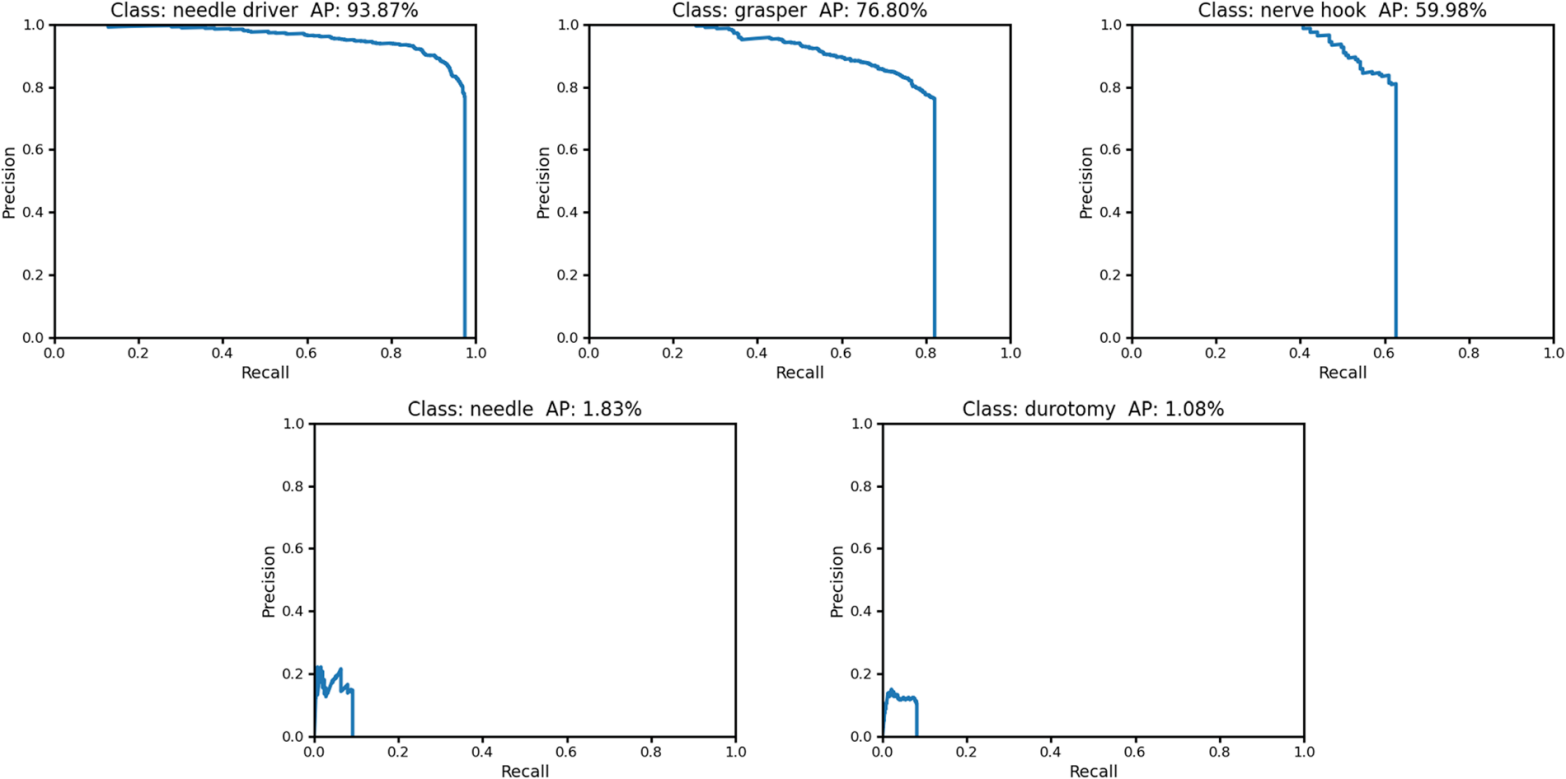
YOLOv4 deep learning instrument detection precision recall curves and average precision. AP, average precision.

**Fig. 4:**
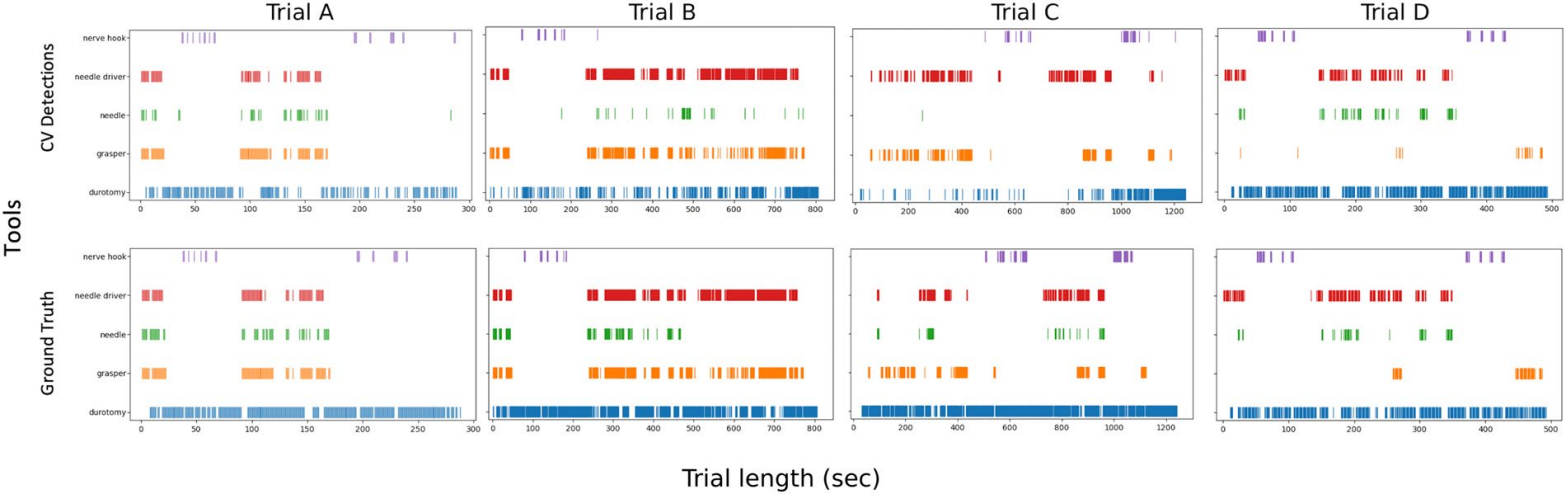
Tool presence comparison between ground-truth and detections for all SOSpine test set trials.

The YOLOv4 computer vision model was trained for object detection with bounding boxes on the SOSpine dataset. 12,866 images from 20 durotomy-repair trials were used for training, 1,000 training images were used for validation, and 2,828 images from 4 trials were held out for testing (Table 1). The overall mean average precision (mAP) for all detected objects was 0.38. The average precision (AP) for detection was 0.939 for the needle driver, 0.768 for the grasper, and 0.600 for the nerve hook (Fig. 3). Detection of needle and durotomy was poor (0.018, 0.011 AP respectively). The mean average precision (mAP) of the instruments (grasper, needle driver, and nerve hook) was 0.77. The training and test splits are shown in Table 1. As seen in Figs. 2, 4, the CV model was able to detect the presence of instruments in a manner that is comparable to ground truth annotations and phases of the durotomy repair procedure.

We chose the YOLOv4 AI model for its public access, and subsequent reproducibility, high performance, and low computational demands. Using this model, we found significant differences in its ability to identify surgical instruments - larger objects with greater depth and higher contrast (e.g., metal instruments such as a grasper) were detected quite well compared to anatomy (i.e., durotomy) or poorly illuminated objects far from the camera source (e.g., needle) (Fig. 3). These findings are consistent with our previous work and facilitate the validation of this video dataset for ML applications^4^.

In the basic sciences, exploratory studies are required prior to larger, more validating works and our benchmark standards serve as a baseline for future model development. Our work also provides insight into expected performance should groups analyze real operative video with similar instruments and viewpoints. We also demonstrate the potential for surgical videos to provide quantitative metrics that reflect surgical outcomes. By analyzing the patterns of instrument usage, we can automatically identify surgical phases by applying procedure-specific rules to the AI-facilitated detection of surgical instruments. For example, the nerve hook and durotomy instruments used together indicate the “knot-tying” phase. Identifying these actions and surgical phases can enable AI systems to offer decision assistance and improve the postoperative review of surgical outcomes and performance^3,4,16,20,22,48–51^. These tools are especially important for “non-routine” events and for developing trainee competency.

In cases where non-routine surgical events occur, a more senior surgeon is likely to take over the primary surgeon role, particularly in the era of reduced reimbursements, duty hour restrictions, and increasingly litigious clientele. However, this approach can limit trainee “repetitions” and delay the achievement of technical competence. Developing AI models that facilitate the management of non-routine events can achieve two goals: improving the trainee’s intraoperative performance, allowing them to remain the primary operator thereby giving them greater autonomy, and generating automatic video review and analysis postoperatively, facilitating the acquisition of technical skills even outside the operating room. With our benchmark results, the SOSpine dataset can be harnessed for further analysis with more advanced statistical tools and automated performance metrics.

Our methodology for dataset validation also has several limitations. In this work, we examined the prediction ability of one off-the-shelf computer vision algorithm on the SOSpine dataset. The SOSpine YOLOv4 CV model is tailored to the task of durotomy repair with a limited set of instruments and may not perform to the same extent in other surgical procedures. Therefore, further statistics analysis, benchmarking, and examination through APMs are needed to use this dataset in a more comprehensive manner. The SOSpine dataset also contains 24 videos of cadaveric simulations and therefore offers a relatively small sample size for both training and testing with CV algorithms. While the videos capture a realistic surgical scenario, there are limitations in the translation of the cadaveric simulation to real intraoperative video, which would entail a less controlled surgical environment. The SOSpine dataset contains video frames from a single institution with only eight unique surgeons, and therefore, our CV model may not generalize to other surgeons or institutions where a spinal durotomy repair is performed differently.

The present spine surgery video dataset release, along with its associated analysis using a computer vision model to identify instruments, demonstrates the feasibility and potential applications of video data in this field. To fully realize the value of video-based AI models, it is crucial to continue developing video datasets and improving surgical object detection, as well as integrating them into the research and clinical workflow. With datasets like SOSpine and further model evaluations, surgical video analysis can become a powerful tool for surgical data scientists, offering new insights and improving patient outcomes.

## Data Availability

The SOSpine dataset was prepared as described in the methods sections with minimal custom processing. Open-source software used for computer vision training and validation can be found on Github (https://github.com/AlexeyAB/darknet). Python code used to generate the figures and perform the technical validation can be found within the FigShare project as described above within the SOSpine.zip file. Python code and necessary dataset files can also be found on GitHub (https://github.com/alanbalugu/SOSpine).
